# The molecular epidemiology of circulating rotaviruses: three-year surveillance in the region of Monastir, Tunisia

**DOI:** 10.1186/1471-2334-11-266

**Published:** 2011-10-03

**Authors:** Mouna Hassine-Zaafrane, Khira Sdiri-Loulizi, Imen Ben Salem, Jérôme Kaplon, Siwar Ayouni, Katia Ambert-Balay, Nabil Sakly, Pierre Pothier, Mahjoub Aouni

**Affiliations:** 1 Laboratory of Infectious Diseases and Biological Agents, Faculty of Pharmacy, University of Monastir, TU-5000 Monastir, Tunisia; 2 National Reference Center for Enteric Viruses, Laboratory of Virology, CHU of Dijon, 2 Rue Angélique Ducoudray, University of Bourgogne, 21000 Dijon, France; 3 Laboratory of Immunology, University Hospital Fattouma Bourguiba, TU-5000 Monastir, Tunisia

## Abstract

**Background:**

Rotavirus infection is the most common cause of severe, dehydrating, gastroenteritis among children worldwide. In developing countries, approximately 1440 children die from rotavirus infections each day, with an estimated 527,000 annually. In infants, rotavirus is estimated to cause more than 2 million hospitalizations every year depending on the income level of the country.

The purpose of this study was to estimate the proportion of rotavirus gastroenteritis and identify the distribution of circulating G and P genotype rotavirus strains among children consulting several dispensaries in the region of Monastir (outpatients departments) or admitted to Monastir University Hospital (inpatients department) with acute gastroenteritis.

**Methods:**

This study was undertaken during a 3-year period from April 2007 to April 2010 in Tunisian children under 13 suffering from acute gastroenteritis. Group A rotaviruses were detected in stools by ELISA and genotyped using multiplex reverse transcription PCRs with type-specific primers on the basis of their outer capsid proteins. Statistical analyses were performed with SPSS software, version 19.

**Results:**

Of the 435 stool samples from children with acute gastroenteritis, 27.6% were positive for rotavirus A. The predominant G type was G1 (37.5%), followed by G3 (25%), G2 (17.5%), G4 (12.5%), G9 (2.5%) and three mixed-G infections G3G4 (2.5%) were identified.

Only P[8] (80.8%), P[4] (16.7%) and P[9] (0.8%) genotypes were found. The predominant single G/P combination was G1P[8] (37.5%), followed by G3P[8] (25%), G2P[4] (16.7%), G4P[8] (12.5%), G9P[8] (1.7%) and one case of the unusual combination G9P[9] (0.8%). The G-mixed types G3G4 combined with P[8] (2.5%). Infants less than 3 months of age were most frequently affected. The prevalence of rotavirus infection peaked in the winter season, when temperatures were low, and decreased in summer.

**Conclusions:**

Rotavirus gastroenteritis is a common disease associated with significant morbidity, mortality, and economic burden. Epidemiological knowledge of rotavirus is critical for the development of effective preventive measures, including vaccines.

These data will help to make informed decisions as to whether rotavirus vaccine should be considered for inclusion in Tunisia's National Immunisation Programme.

## Background

Group A rotaviruses are the most commonly detected viral cause of severe diarrhoea in infants and young children worldwide, infecting more than 125 million infants and young children every year, causing an estimated 527,000 deaths [1].

They are members of the *Reoviridae *family and contain a genome consisting of 11 segments of double stranded RNA (dsRNA) enclosed in a triple layered capsid [2]. The outer layer of rotaviruses is composed of two proteins, VP7 and VP4, encoded by RNA segments 9 and 4, respectively. Those proteins elicit neutralising antibody responses and form the basis of the current classification of group A rotaviruses into G (VP7) and P (VP4) types, where G stands for glycoprotein and P for protease sensitive protein [3]. Currently, 27 G genotypes and 35 P genotypes have been reported in humans and animals [4]. A dual typing system is necessary in order to characterise the strains of rotavirus co-circulating during different rotavirus seasons in different locations. Globally, P[8], P[4] and G1 to G4 and G9 are the most common P- and G-types in humans. However, a limited number of combined G/P genotypes are common in humans, such as G1P[8], G2P[4], G3P[8], G4P[8], and G9P[8] [5], with G1P[8] as the most prevalent rotavirus type detected in most surveys. Nevertheless, other G- and P types have been found to be highly prevalent in different areas of the world (e.g. G5 types in Brazil, G10 types in India) [6,7], and more recently, the combinations G12P[8] and G12P[6] have been reported [8].

Assessing the impact of a rotavirus vaccine should take into account the natural temporal variability in P and G types. The compositions of the two live oral rotavirus vaccines, Rotarix^® ^and Rotateq™, present very different immunization strategies, and seem to provide cross-protection against rotavirus diarrhoea caused by multiple serotypes. While both have demonstrated safety and proved to be efficacious in various developed and developing country settings, vaccine effectiveness following implementation will have to be closely monitored. In this way, any influence on vaccine efficacy by the emergence and temporal increase in the frequency of new variants, both naturally occurring and induced through the process of genotype replacement, can be determined. Longitudinal genetic surveillance of disease causing rotaviruses is a clear priority, and particularly so in Africa prior to and after wide scale vaccine introduction.

This study summarizes epidemiological data for rotavirus infections collected over 3 years (2007-2010) from Tunisian children hospitalized or presented to dispensaries of Monastir. To complement knowledge on rotavirus infections in this country, we described the proportion, the seasonality and the genotypic characteristics of rotavirus strains responsible for acute gastroenteritis in children. In Tunisia, it will be important to maintain ongoing rotavirus surveillance to understand the distribution of G and P genotypes in order to monitor the impact of rotavirus vaccines once they have been introduced into the immunization schedule for infants.

## Methods

From April 2007 to April 2010, a total of 435 stool specimens were collected from children (239 males and 196 females) under 13 years of age. Among them 238 stool samples were collected from inpatients within 48 h following their hospitalization for acute gastroenteritis in Monastir University Hospital. One hundred ninety seven samples were also collected from outpatients (non-hospitalized) consulting several dispensaries in the region of Monastir for gastrointestinal symptoms. Children with nosocomial or chronic diarrhoeas were excluded from the study.

All the patients and their parents were informed by the doctors about the study and they agreed to participate by giving stool samples and informed the doctors about any needed information. This study did not require approval or review by our institutional review board.

The stool samples were routinely screened for the presence of VP6 group A rotavirus antigen by enzyme immunoassay using a commercial ELISA kit (Rotavirus ELISA kit, Premier™ Rotaclone^®^). All rotavirus-positive samples were confirmed by RT-PCR and genetically characterized. The viral RNA was extracted from 20% stool suspensions in phosphate-buffered saline using the NucliSENS^® ^EasyMAG™ platform (bioMérieux, Marcy L'Etoile, France), according to the manufacturer's instructions. Rotavirus G and P genotyping was performed using semi-nested type specific multiplex RT-PCRs that could detect seven G-types and six P-types. Briefly, in order to identify the G type, an 881 nucleotide fragment of the VP7 region was amplified with generic primers VP7F and VP7R [9]. The G genotypes were subsequently determined on amplicons obtained using a pool of internal primers specific for G1-G4 and G8-G10 rotavirus genotypes in combination with the appropriate reverse consensus primers [9]. Similarly, a 663 nucleotide fragment of the VP4 region was reverse transcribed and amplified using the generic primers VP4F and VP4R, and P genotyping was done using internal primers specific for P[4], P[6], P[8], P[9], P[10] and P[11] [10]. All the RT-PCRs were performed with viral RNA extracted from reference samples as positive controls and RNase free water as negative control. RT-PCR fragments were electrophoresed in 2% agarose gels in Tris-borate-EDTA buffer along with a 50-bp DNA ladder (Invitrogen) as a standard marker. The gels were stained with ethidium bromide and amplicons were viewed with UV light.

Statistical analyses were performed with SPSS^® ^software, version 19. We compared categorical data by the chi-square (X^2^) test. All data are expressed as means ± standard deviations (SD). Student's *t *test was used to compare means between qualitative data and one-way ANOVA to compare means between quantitative data. Correlations between qualitative variables were assessed using Spearman's correlation coefficient. Statistical analyses using X^2 ^or Fisher's exact test were performed for comparisons of percentages. All tests were two-tailed; P values ≤ 0.05 were considered statistically significant.

## Results

Of the 435 faecal samples analysed, 123 tested positive for rotavirus by ELISA. Of these, only 3 samples were not amplifiable by RT-PCR. Group A rotaviruses were identified in 120 (27.6%) stool samples. Seven (1.6%) noroviruses were detected as mixed infections with rotavirus, 3 in hospitalized children and 4 in non-hospitalized children.There was no significant difference between the rotavirus infections in hospitalized (26.5%) and in non-hospitalized (28.9%) children (P = 0.567). A total of 72 males (30.1%) and 48 females (24.5%) were rotavirus positive.

### Age and seasonal distribution

**Figure 1:**
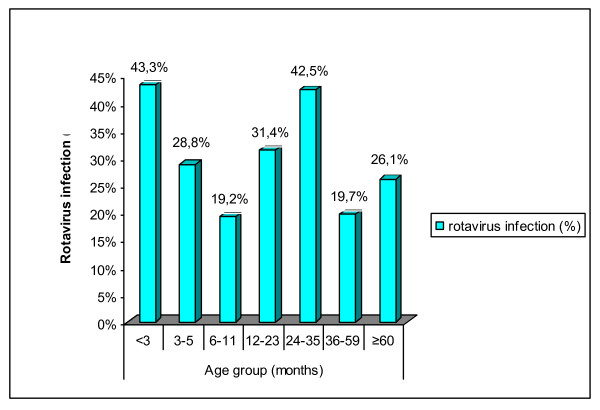
**Age distribution of rotavirus infections among children younger than 13 years**.

The age distribution of children with acute rotavirus diarrhoea as diagnosed by multiplex RT-PCR is depicted in Figure 1. Ages ranged from 18 days to 156 months, with a mean of 19.8 ± 20.19 months and a median of 12 months, and there were no significant differences among the results obtained for rotavirus with regard to mean and median ages. The most common age group was < 3 months (43.3%), followed by 24-35 months age group (42.5%), 12-23 months age group (31.4%), 3-5 months age group (28.8%), ≥ 60 months age group (26.1%), 36-59 months age group (19.7%) and 6-11 months age group (19.2%). Overall, 85% of rotavirus positive samples were from children less than 3 years old but there was no significant difference (P = 0.150) between the 2 age groups regarding the prevalence of rotavirus A (29.3% in children under 3 years and 21.4% in children 3 years or more).

**Figure 2:**
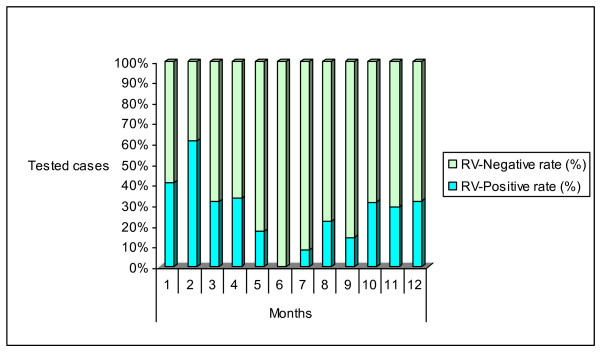
**Monthly distribution of rotavirus infections among children younger than 13 years**.

Rotavirus was detected throughout the study period but there was a distinct seasonality of rotavirus diarrhoea. The peak detection of rotavirus occurred during the months of the cool dry season, with 41% and 61% rotavirus-positive samples in January and February, respectively, with low prevalence in June and July (Figure 2). Rotavirus prevalence was 38.6% in winter, 26.3% in spring, 26.1% in autumn and 14.1% in summer. There was a significant difference in the seasonal distribution (P = 0.01). This global distribution over the 3- year study period was also observed for each separate year. There is no change in the seasonal distribution over years.

### Distribution of G and P genotypes

A total of 120 rotavirus-positive samples were G and P genotyped (Table 1). Whereas 117 (97.5%) faecal specimens contained only one rotavirus strain, 3 (2.5%) were mixed rotavirus infections. Overall, during the study, 37.5% of strains identified were genotype G1, 25% G3, 17.5% G2, 12.5% G4, 2.5% G9 and 2.5% G3G4. One strain was G not typeable. Of these rotavirus strains, 97 were characterized as P[8] (80.8%), 20 as P[4] (16.7%), 1 as P[9] (0.8%), and 2 were P non-typeable (1.7%).

**Table 1:** Distribution and detection rate of G and P genotype combinations of rotavirus detected from 2007 to 2010 in Tunisian children

Genotype combinations	Number of strains (detection rate %)		
	2007-2008	2008-2009	2009-2010
**Common strains**			
G1P[8]	11 (26.8%)	13 (32.5%)	21 (55.3%)
G2P[4]	3 (7.3%)	2 (5%)	15 (39.5%)
G3P[8]	11 (26.8%)	18 (45%)	1 (2.6%)
G4P[8]	10 (24.4%)	5 (12.5%)	0 (0%)
G9P[8]	2 (4.9%)	0 (0%)	0 (0%)
**Unusual strains**			
G9P[9]	1 (2.4%)	0 (0%)	0 (0%)
**Other**			
Partially typed	0 (0%)	2 (5%)	1 (2.6%)
Mixed infections	3 (7.3%)	0 (0%)	0 (0%)

The predominant single G/P combination was G1P[8] (37.5%), followed by G3P[8] (25%), G2P[4] (16.7%), G4P[8] (12.5%), G9P[8] (1.7%) and one case of the unusual combination G9P[9] (0.8%). The G-mixed types combined with P[8]: G3G4P[8] (2.5%). Among 2 G2 strains, the P type could not be determined whereas in one P[8] strain, the G type remained untypeable. There were no significant differences in the distribution of rotavirus genotypes between males and females, or between hospitalized and non-hospitalized children.

Over the three seasons of the study, the prevalence of rotavirus infection was similar: 34.2% in 2007-2008, 33.3% in 2008-2009 and 32.5% in 2009-2010. Interestingly, the number of G1P[8] strains increased from 26.8% in the season 2007-2008 to 55.3% in 2009-2010, whereas G2P[4] increased from 7.3% in 2007-2008 to 39.5% in 2009-2010, G3P[8] decreased from 26.8% to 2.6% and G4P[8] decreased from 24.4% in 2007-2008 to 12.5% in 2008-2009. Regarding G9P[8], G9P[9] and G3G4P[8], these genotypes appeared only in the 2007-2008 season (Table 1).

## Discussion

The present study described the epidemiology of rotavirus infections in hospitalized and non-hospitalized children and infants in various towns of the region of Monastir between 2007 and 2010. Rotavirus is the most common cause of non-bacterial gastroenteritis in children, not only in developing countries but also in developed countries. A total of 435 faecal specimens were tested for rotavirus and 27.6% were positive. These results are consistent with previous findings on rotavirus prevalence in Tunisia (21% and 22.5%) [11,12]. Similar proportions (16% to 23%) of rotavirus gastroenteritis were found in different Arab countries like Saudi Arabia [13-17] and Egypt [18-21], but this is lower than the prevalence of rotavirus attained in Syria (61%) [19], Oman (50%) [22] and Kuwait (44%) [23]. These different detection rates may be explained by different conditions of the studies, such as the season of sampling and the sampling methods. For example, in other studies [24], samples were collected only from hospitalized children, whereas in our study, they were collected from inpatients and outpatients, which may have affected the prevalence rates.

Rotavirus was detected continuously throughout the years of study with the exception of June, with peak prevalence occurring in February. Interestingly a similar study conducted by Sdiri et al. from January 2003 to April 2007 [12 and unpublished observations] demonstrated that in Tunisia rotavirus infections peak during the colder months of the year and from June to September. A previous study in Sousse (Tunisia) [25] showed that rotavirus infection was found to occur predominantly in the cooler season, with most cases occurring between September and January with the peak in November. The change in the temporal distribution of rotavirus cases can be explained by the variability of mean temperature, relative humidity, and rainfall. A number of other Arab countries including Iran [26-29], Libya [30], Morocco [31], Oman [22], and Saudi Arabia [14] also reported that the peak season for rotavirus gastroenteritis was in the winter from November to April. The exception to this is Egypt, where rotavirus infections peak from July to November [20,32].

One of the main goals of this study was to characterize the VP7 (G genotype) and VP4 (P genotype) gene segments of the Tunisian rotavirus strains. We identified most of the common rotavirus combinations in our study. G1P[8] was identified with a high prevalence (37.5%), followed by G3P[8] (25%), G2P[4] (16.7%), G4P[8] (12.5%), G3G4P[8] (2.5%), G9P[8] (1.7%) and one case of the unusual combination G9P[9] (0.8%).

The G1P[8] genotype combination was the most prevalent rotavirus type observed in Tunisia from 1995 to 2004 [33], the G3P[8] was the predominant combination detected during 2005 to 2006 [11]. In our study from 2007 to 2010 strains bearing G1P[8] antigens were predominant. This variability in the genotype profiles has been shown in previous studies to be dependent on a given place and a period of time [13,18,28,32].

It has to be mentioned that this study dealt with strains isolated from only one city in Tunisia (in and around the region of Monastir) and surveillance was limited to a period of 3 years. It will be important to continue surveillance and the characterization of rotavirus strains in Monastir in order to monitor changes over time. It is equally important to initiate studies in other regions of Tunisia in order to have a comprehensive picture of strain distribution in the country.

## Conclusions

In conclusion, the present study confirms the current burden of rotavirus gastroenteritis in younger children, especially small infants, and highlights the diversity of rotavirus strains circulating in Monastir and neighboring areas. Continuous prospective monitoring of circulating strains of rotavirus is desirable to detect any changes in their distribution promptly and to assess the effectiveness of active immunization programs. Future studies are needed in which systematic surveillance of gastroenteritis is done for long periods of time with harmonized methods in order to find explanations for the apparent emergence of rotavirus variants in populations.

## Competing interests

The authors have no commercial disclosures to make with regard to this manuscript. No competing financial interests exist.

## Authors' contributions

MHZ initiated and designed the study and was responsible for the collection of specimens and clinical information as well as data analysis, molecular studies, genotyping of rotavirus and preparation of the manuscript. KSL carried out the analysis and interpretation of data and prepared the manuscript. IBS collected stools from dispensaries and carried out the microbiological analysis. JK participated in the design of the methodology and contributed to the interpretation of results. SA helped in the collection of 21 stools from dispensaries. KAB helped draft the manuscript and was involved in revising the manuscript critically for intellectual content. NS performed the statistical analysis. PP initiated the study, and participated in its design and coordination. MA initiated the study, and participated in its design and coordination. All authors read and approved the final manuscript.

## Pre-publication history

The pre-publication history for this paper can be accessed here:

http://www.biomedcentral.com/1471-2334/11/266/prepub
